# Supplementary material for: The Microtubule-Targeting Agent Pretubulysin Impairs the Inflammatory Response in Endothelial Cells by a JNK-Dependent Deregulation of the Histone Acetyltransferase Brd4
Source: Cells. 2023 Aug 21;12(16):2112. doi: 10.3390/cells12162112 (PMC10453553; doi:10.3390/cells12162112)
Supplement: Supplementary file 1 [file cells-12-02112-s001.zip › cells-2531440-supplementary.pdf]

The Microtubule-Targeting Agent Pretubulysin impairs the Inflammatory Response in Endothelial Cells by a JNK-dependent Deregulation of the Histone Acetyltransferase Brd4

Supplementary Materials

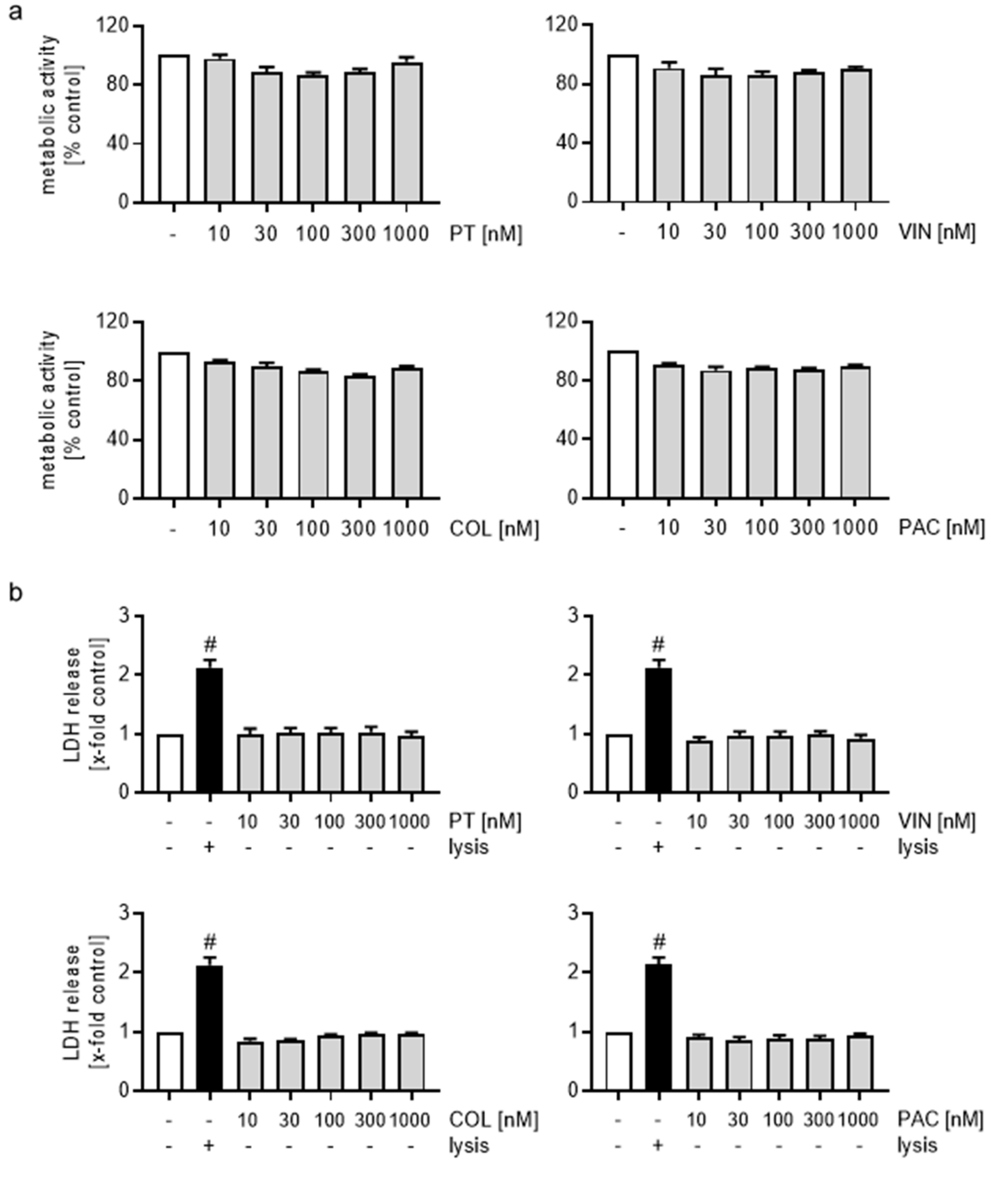

Supplemental Figure S1. Pretubulysin and the other MTAs do not significantly impact the metabolic activity and membrane integrity of HUVECs. Confluent HUVECs were treated with different concentrations (10, 30, 100, 300, 1000 nM) of pretubulysin (PT), vincristine (VIN), colchicine (COL) or paclitaxel (PAC) for 24 h. (**a**) Metabolic activity measured by fluorescence reading and (**b**) the membrane integrity as measured by absorbance reading. For control, cells were lysed with detergent (n = 4). Data are expressed as mean ± SEM. # $p \leq 0.05$ versus control, * $p \leq 0.05$ versus TNF control.

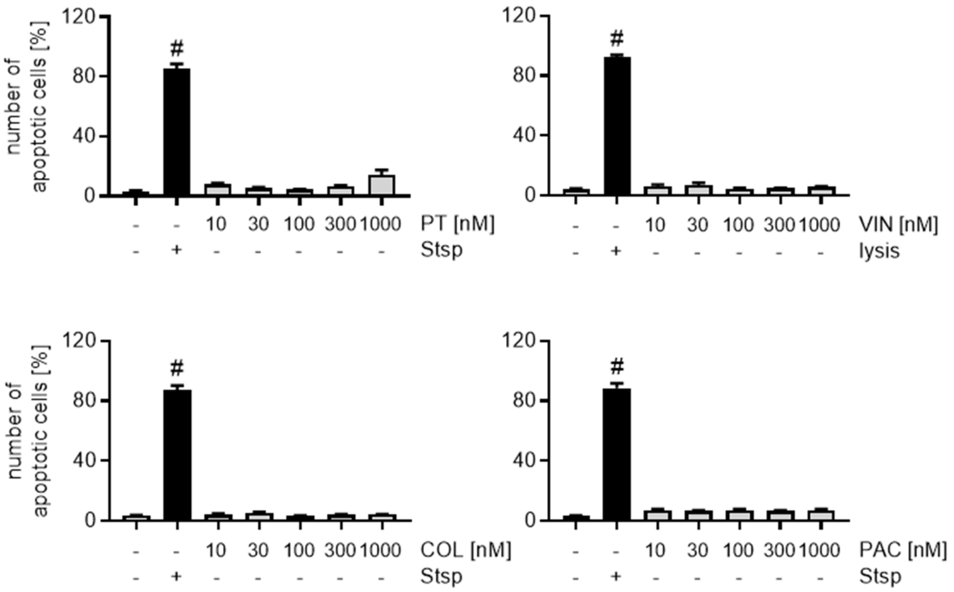

Supplemental Figure S2. MTAs do not significantly increase apoptosis in HUVECs. Flow-cytometric analysis of the number of sub-diploidic cells. Confluent HUVECs were treated with different concentrations (10, 30, 100, 300, 1000 nM) of pretubulysin (PT), vincristine (VIN), colchicine (COL) or paclitaxel (PAC) for 24 h or were treated with 10 μM staurosporine for 24 h. (n = 4) Data are expressed as mean ± SEM. # $p \leq 0.05$ versus control.

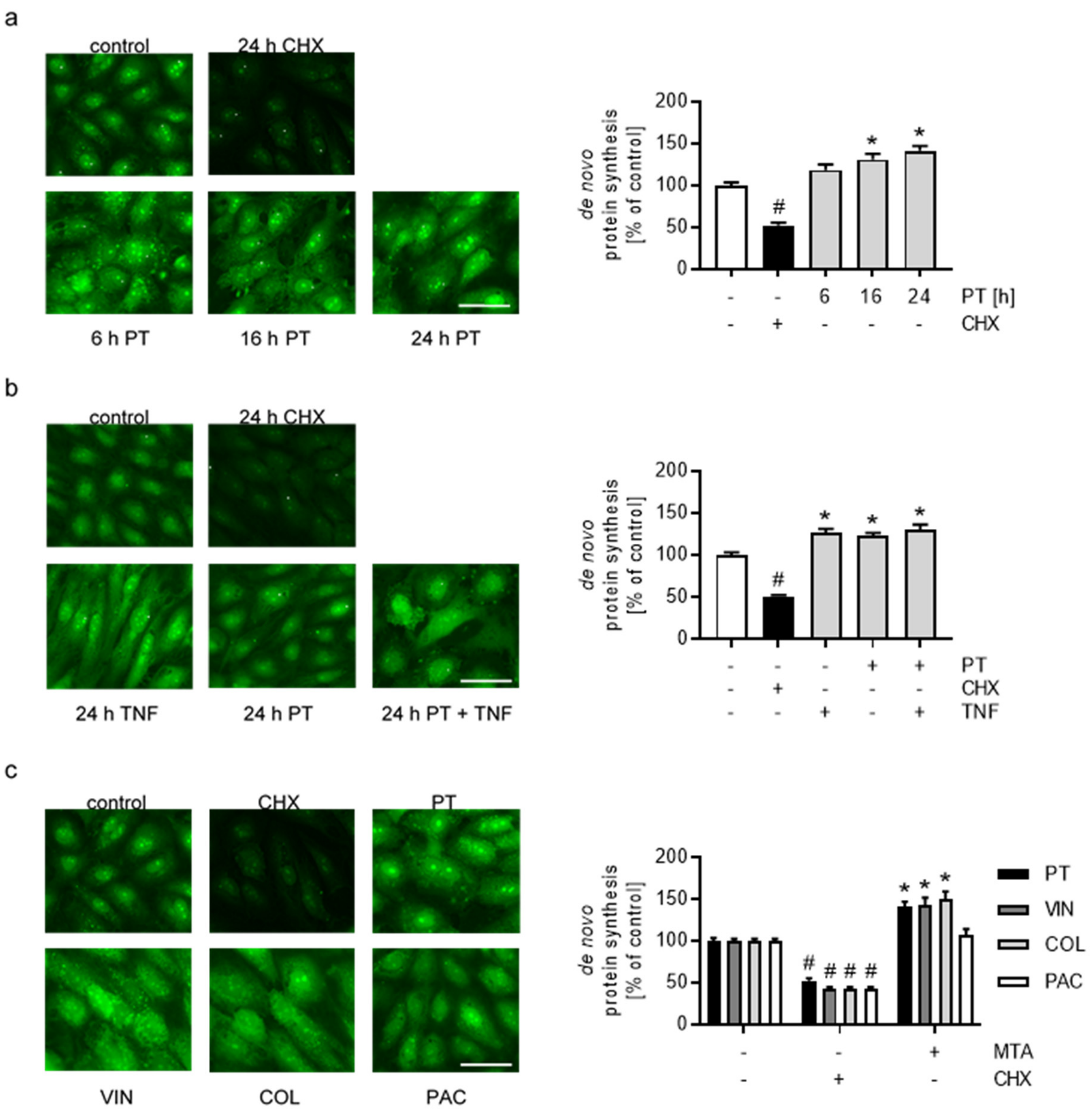

Supplemental Figure S3. Pretubulysin and the other MTAs do not negatively impact the *de novo* protein synthesis. Microscopical analysis of *de novo* protein synthesis via immunostaining of HUVECs. Confluent HUVECS were treated with pretubulysin (PT) for different durations (6, 16, 24 h; a) or pre-treated with PT for 30 min and/or activated with TNF (10 ng/mL) for 24 h (b) or treated with PT, vincristine (VIN), colchicine (COL) or paclitaxel (PAC) for 24 h (c). 24 h of cycloheximide (1 µg/mL) treatment was used as positive control. (n = 3) Data are expressed as mean ± SEM. # $p \leq 0.05$ versus control, * $p \leq 0.05$ versus TNF control. The bar represents 20 µm distance.

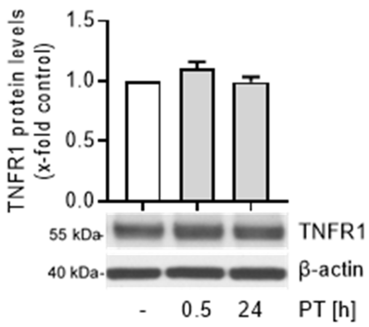

Supplemental Figure S4. Pretubulysin does not affect total TNFR1 protein levels. Western blot analysis of the TNFR1 protein levels. Confluent HUVECs were treated with pretubulysin (PT; 300 nM) for 0.5 h or 24 h. Results were normalized on the respective β-actin levels. (n = 3)

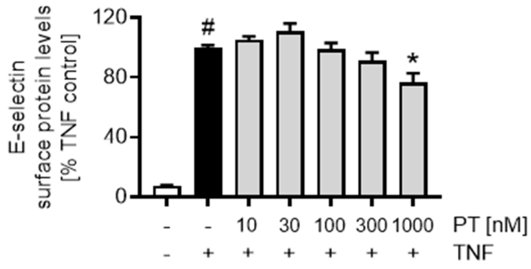

Supplemental Figure S5. Pretubulysin only slightly decreases the E-selectin cell surface levels. Flow-cytometric analysis of the E-selectin cell surface levels. Confluent HUVECs were pre-treated with different concentrations of pretubulysin (PT; 10, 30, 100, 300, 1000 nM for 20 h and activated with TNF (10 ng/mL) for 4 h. (n ≥ 3) Data are expressed as mean ± SEM. # $p \leq 0.05$ versus control, * $p \leq 0.05$ versus TNF control.

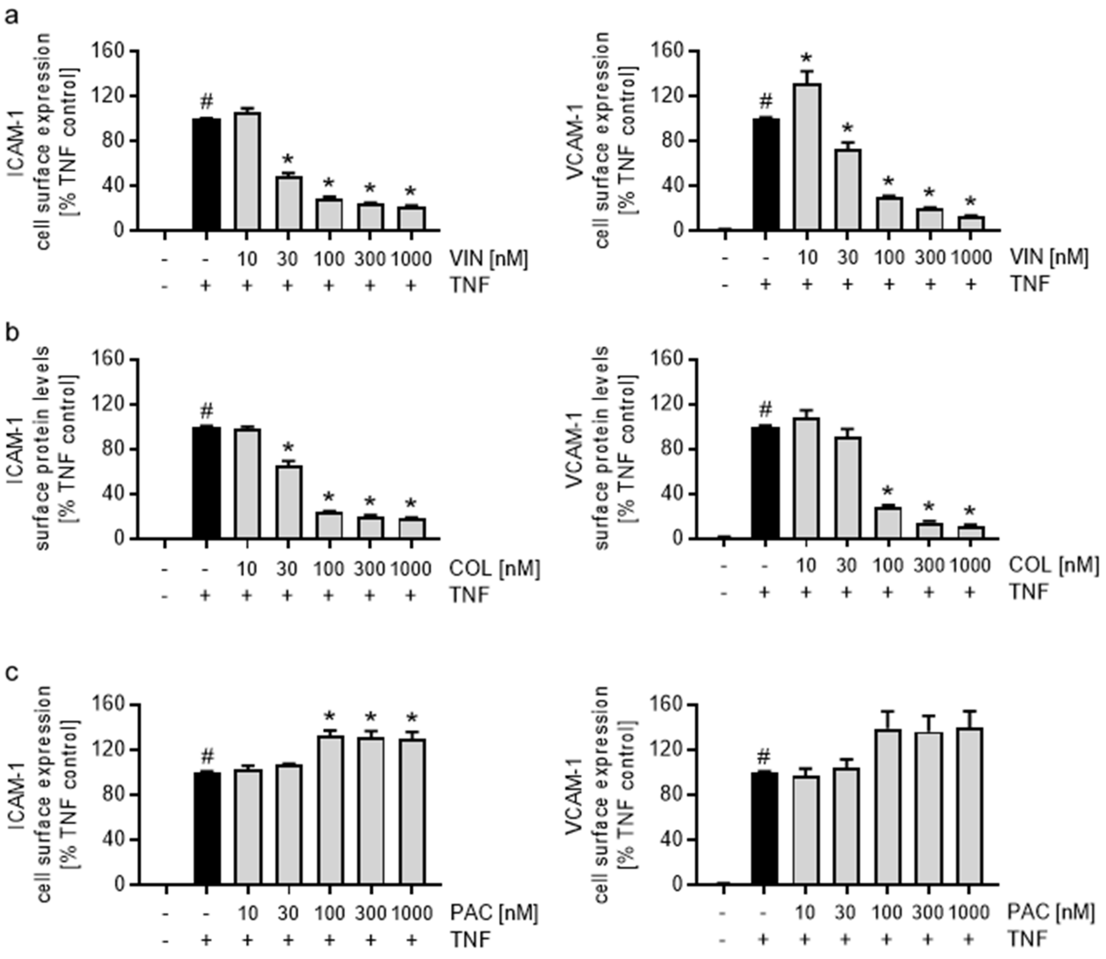

Supplemental Figure S6. The depolymerizing MTAs decrease the cell surface levels of ICAM-1 and VCAM-1 in a concentration-dependent manner. Flow cytometric analysis of the cell surface levels of ICAM-1 (a, b, c; left) and VCAM-1 (a, b, c; right) after treatment with depolymerizing or stabilizing MTAs. Confluent HUVECs were pre-treated with different concentrations (10, 30, 100, 300, 1000 nM) of vincristine (VIN), colchicine (COL) or paclitaxel (PAC) for 30 min and activated with TNF (10 ng/mL) for 24 h. (n = 3) Data are expressed as mean ± SEM. # $p \leq 0.05$ versus control, * $p \leq 0.05$ versus TNF control.

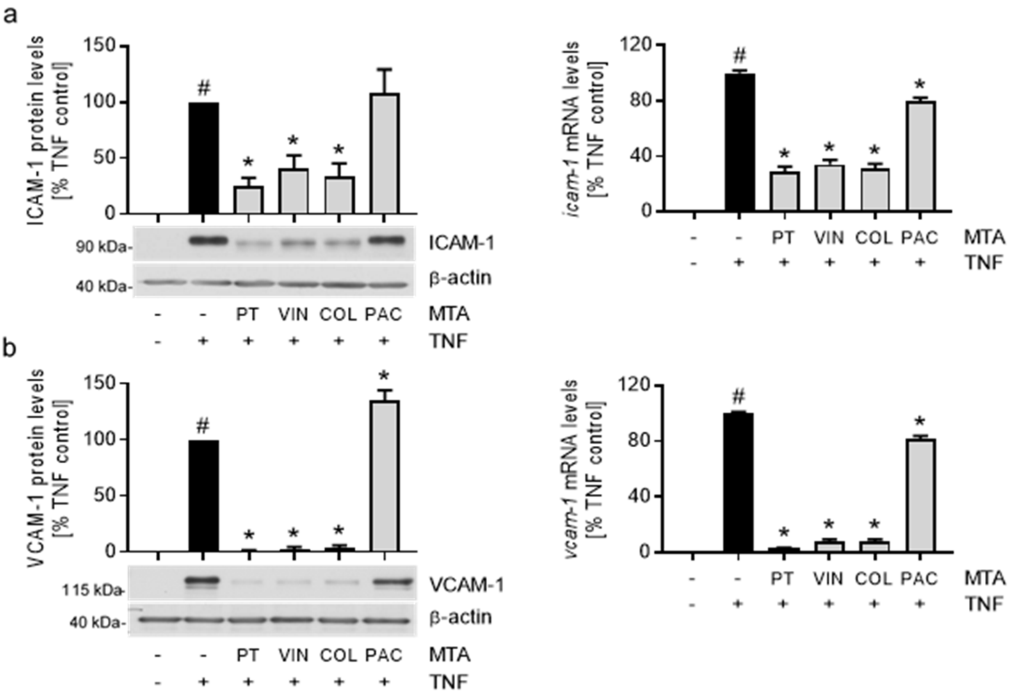

Supplemental Figure S7. The destabilizing MTAs reduce the protein levels and mRNA levels of ICAM-1 and VCAM-1. Western blot analysis of the total ICAM-1 and VCAM-1 protein levels (a and b, left) and qPCR analysis of the mRNA levels of ICAM-1 and VCAM-1 (a and b, right). Confluent HUVECs were pre-treated with 300 nM of pretubulysin (PT), vincristine (VIN), colchicine (COL) or paclitaxel (PAC) and activated with TNF (10 ng/mL) for 24 h (a and b, left) or 12 h (a and b, right). Western blot results were normalized on the respective β-actin and qPCR results were normalized on the respective *gapdh* levels. (n = 3) Data are expressed as mean ± SEM. # $p \leq 0.05$ versus control, * $p \leq 0.05$ versus TNF control.

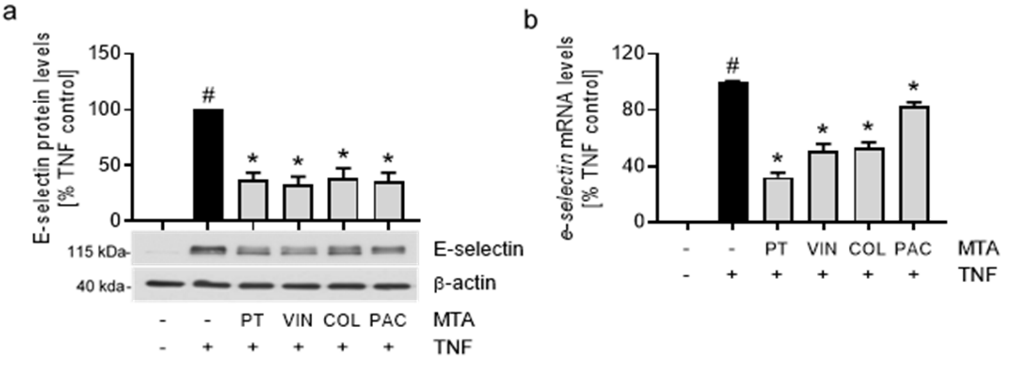

Supplemental Figure S8. The depolymerizing MTAs significantly decrease the E-selectin total protein and mRNA levels. (a) Western blot analysis of the total E-selectin protein levels and (b) *e-selectin* mRNA levels. Confluent HUVECs were pre-treated with 300 nM of pretubulysin (PT), vincristine (VIN), colchicine (COL) or paclitaxel (PAC; b) for 20 h and activated with TNF (10 ng/mL) for 4 h (a) or pre-treated with 300 nM of PT, VIN, COL or PAC for 10 h and activated with TNF (10 ng/mL) for 2 h (b). Western blot results were normalized on the respective β-actin and qPCR results were normalized on the respective *gapdh* levels. (n = 3) Data are expressed as mean ± SEM. # $p \leq 0.05$ versus control, * $p \leq 0.05$ versus TNF control.

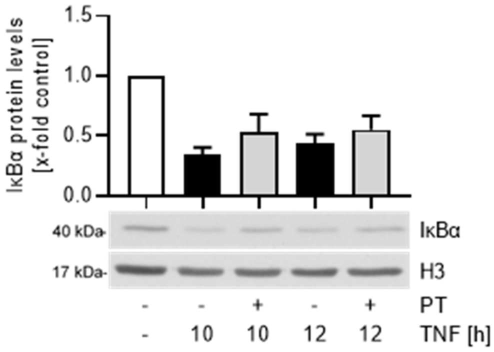

Supplemental Figure S9. Pretubulysin increases the IκBα total protein levels. Western blot analysis of the IκBα protein levels. Confluent HUVECs were pre-treated with pretubulysin (PT; 300 nM) for 30 min and activated with TNF (10 ng/mL) for 10 or 12 h. Results were normalized on the respective histone 3 (H3) levels. (n = 3)

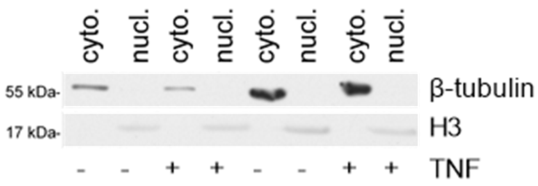

Supplemental Figure S10. Qualitative western blot analysis of the purity of cytoplasmic and nuclear fractions for western blot of cell fractions based on the presence of the cytoplasmic marker β-tubulin and the nuclear marker histone 3. Confluent HUVECs were treated with TNF (10 ng/mL) for 2 h.

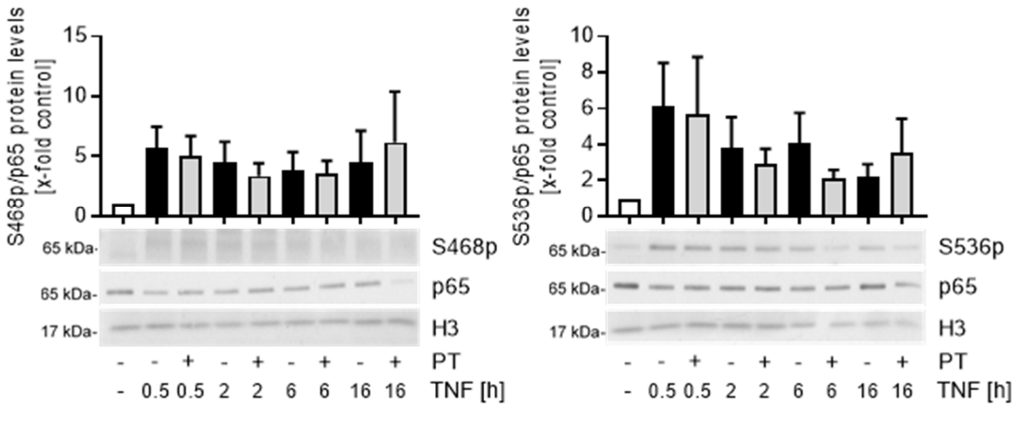

Supplemental Figure S11. Pretubulysin has no significant influence on the NFκB p65-S468 (left) and p65-S536 (right) phosphorylation levels. Confluent HUVECs were pre-treated with pretubulysin (PT; 300 nM) for 30 min and activated with TNF (10 ng/mL) for different duration (0.5, 2, 6, 16 h). Results were normalized on the respective histone 3 (H3) levels. (n = 3)

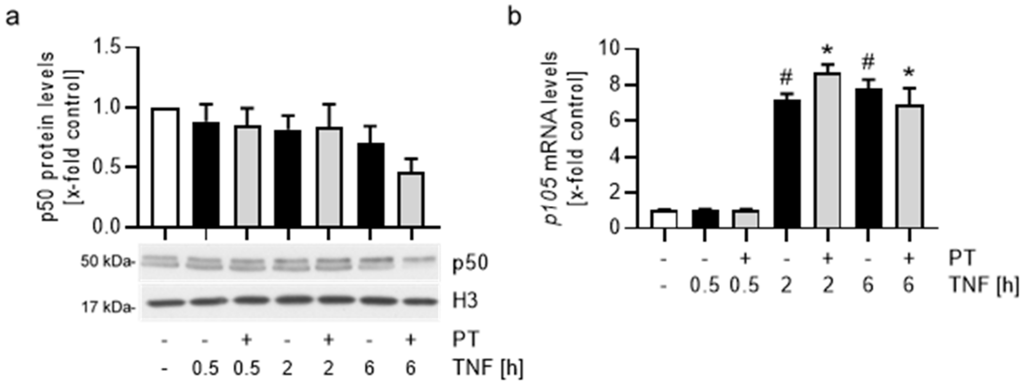

Supplemental Figure S12. Pretubulysin negatively influences the total NFκB-p50 levels. Western blot analysis of the total p50 protein levels and (a) qPCR analysis of the *p105* mRNA levels (b). Confluent HUVECs were pre-treated with pretubulysin (PT; 300 nM) for 30 min and activated with TNF (10 ng/mL) for different duration (0.5, 2, 6 h). Results were normalized on the respective histone 3 (H3; a) or *gapdh* levels (b). (n = 3) Data are expressed as mean ± SEM. # $p \leq 0.05$ versus control, * $p \leq 0.05$ versus TNF control.

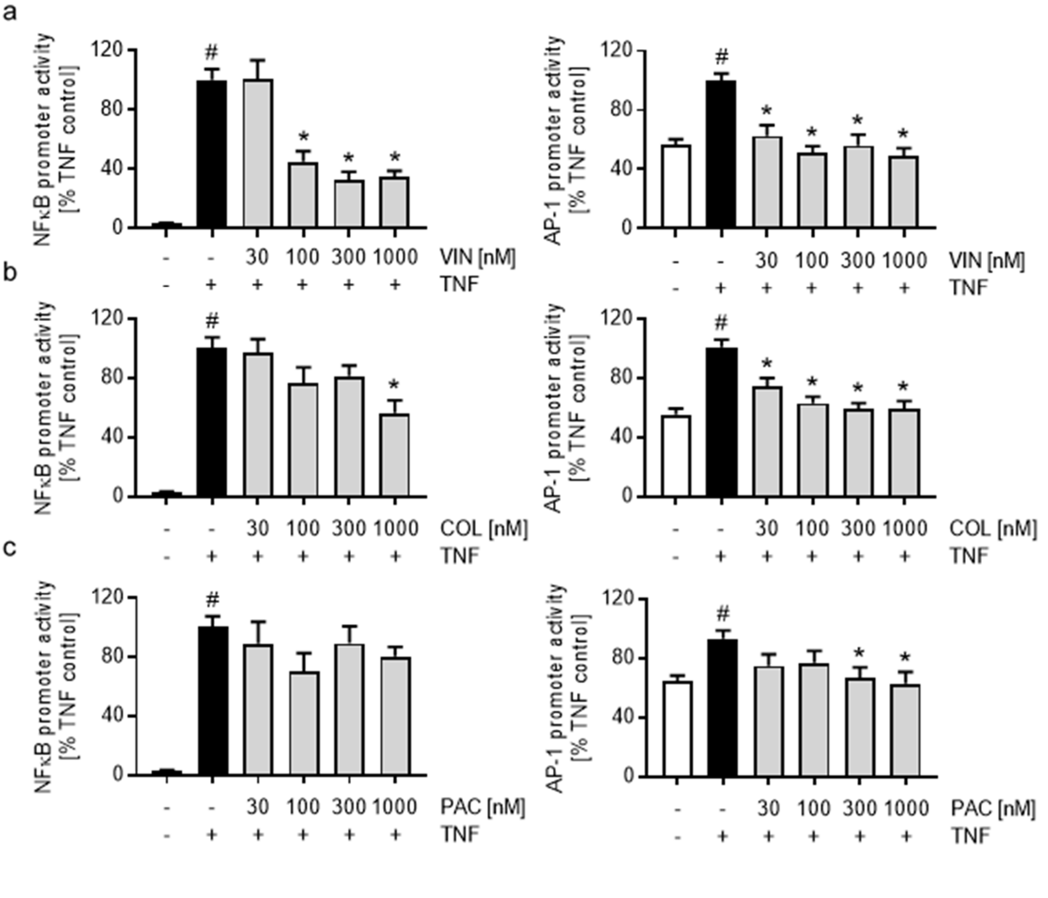

Supplemental Figure S13. MTAs decrease the NFκB and AP-1 promoter activity. Reporter gene assay of the NFκB (a, b, c; left) or AP-1 (a, b, c; right) promoter activity. HUVECs were transfected with pGL4.32[*luc2p*/NF-κB-RE/Hygro] or pGL4.44[*luc2p*/AP-1-RE/Hygro] and pGL4.74[*hRluc*/TK] for normalization and were pre-treated with different concentrations (30, 100, 300, 1000 nM) of vincristine (VIN), colchicine (COL) or paclitaxel (PAC) for 30 min and then activated with TNF (10 ng/mL) for 6 h. (n = 3) Data are expressed as mean ± SEM. # $p \leq 0.05$ versus control, * $p \leq 0.05$ versus TNF control.

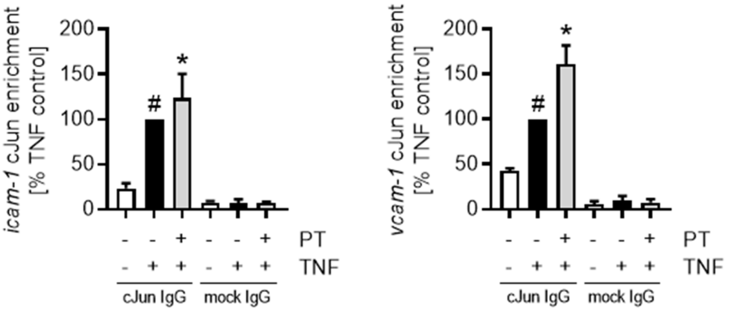

Supplemental Figure S14. Pretubulysin induces the AP-1 cjun enrichment at the *icam-1* and *vcam-1* promoter. Chromatin immunoprecipitation (ChIP) of the AP-1 cJun enrichment at the *icam-1* (left) and *vcam-1* (right) promoters. Confluent HUVECs were pre-treated with pretubulysin (PT; 300 nM) and activated with TNF (10 ng/mL) for 2 h. ChIP was performed with an antibody against cJun and the respective mock antibody. Results were normalized on the background levels of a sequence of the *rpl13a* 3'-untranslated region. (n = 3) Data are expressed as mean ± SEM. # $p \leq 0.05$ versus control, * $p \leq 0.05$ versus TNF control.

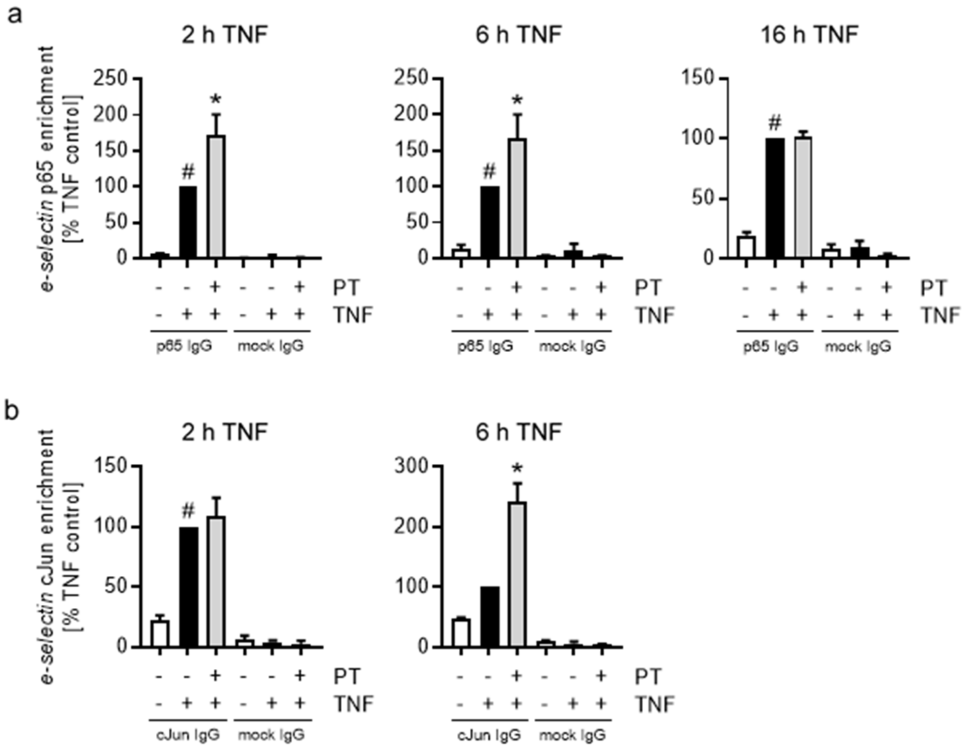

Supplemental Figure S15. Pretubulysin induces the NFκB-p65 and AP-1-cJun enrichment at the *e-selectin* promoter. Chromatin immunoprecipitation (ChIP) of the NFκB-p65 (a) and AP-1-cJun (b) enrichment at the *e-selectin* promoter. Confluent HUVECs were pre-treated with 300 nM of pretubulysin (PT) and activated with TNF (10 ng/mL) for different durations (2, 6, 16 h; a or 2, 6 h; b). ChIP was performed with an antibody against p65 or cJun and the respective mock antibody. Results were normalized on the background levels of a sequence of the *rpl13a* 3'-untranslated region. (n = 3) Data are expressed as mean ± SEM. # $p \leq 0.05$ versus control, * $p \leq 0.05$ versus TNF control.

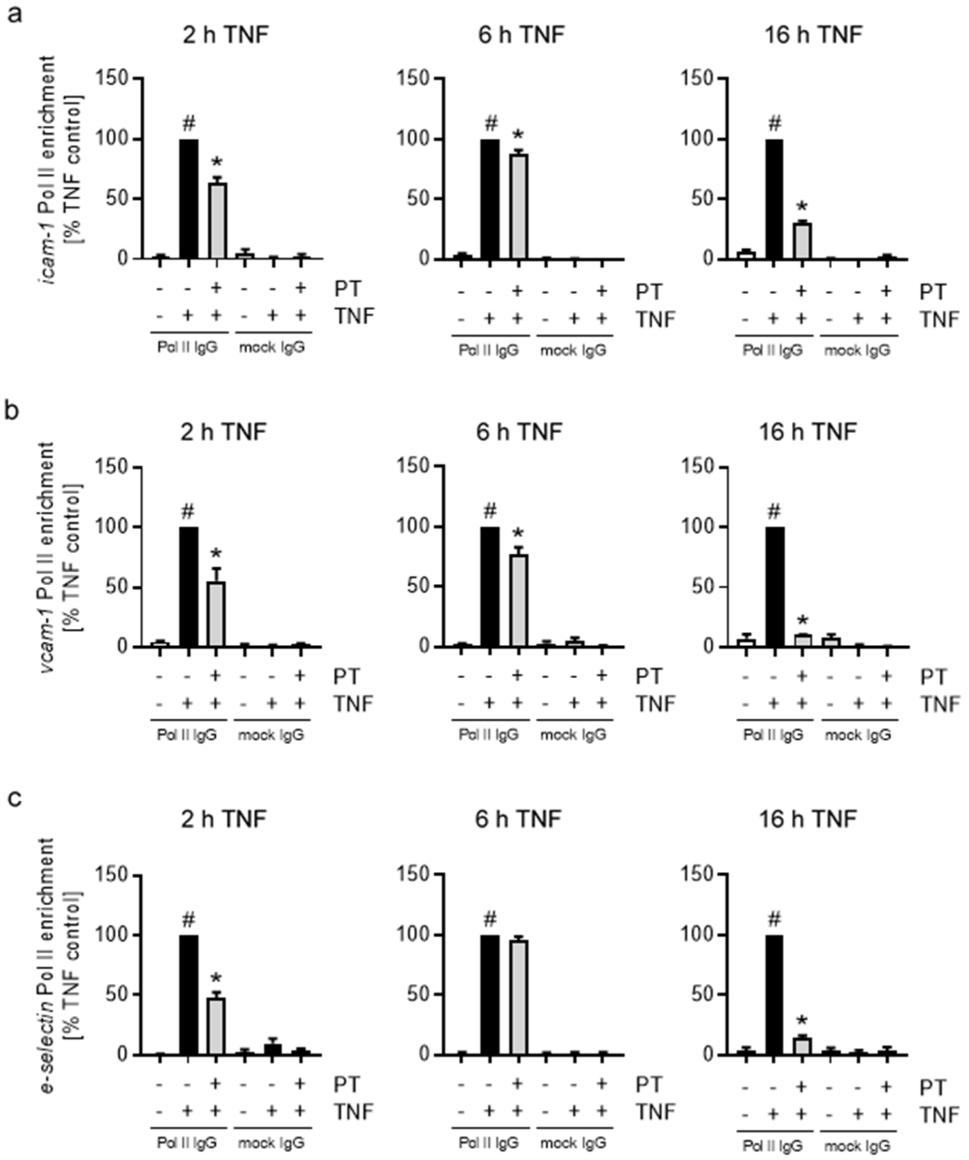

Supplemental Figure S16. Pretubulysin decreases the RNA polymerase II enrichment at the *icam-1, vcam-1* and *e-selectin* promoter in a time-dependent manner. Enrichment of the RNA polymerase II in the promoters of *icam-1* (a), *vcam-*1 (b) and *e-selectin* (c) as shown by chromatin immunoprecipitation (ChIP). Confluent HUVECs were pre-treated with PT (300 nM) for 30 min and activated with TNF (10 ng/mL) for different durations (2, 6, 16 h). ChIP was performed with an RNA II polymerase antibody and the respective IgG mock antibody. Results were normalized on the background levels of a sequence of the *rpl13a* 3'-untranslated region. (n = 3) Data are expressed as mean ± SEM. # $p \le 0.05$ versus control, * $p \le 0.05$ versus TNF control.

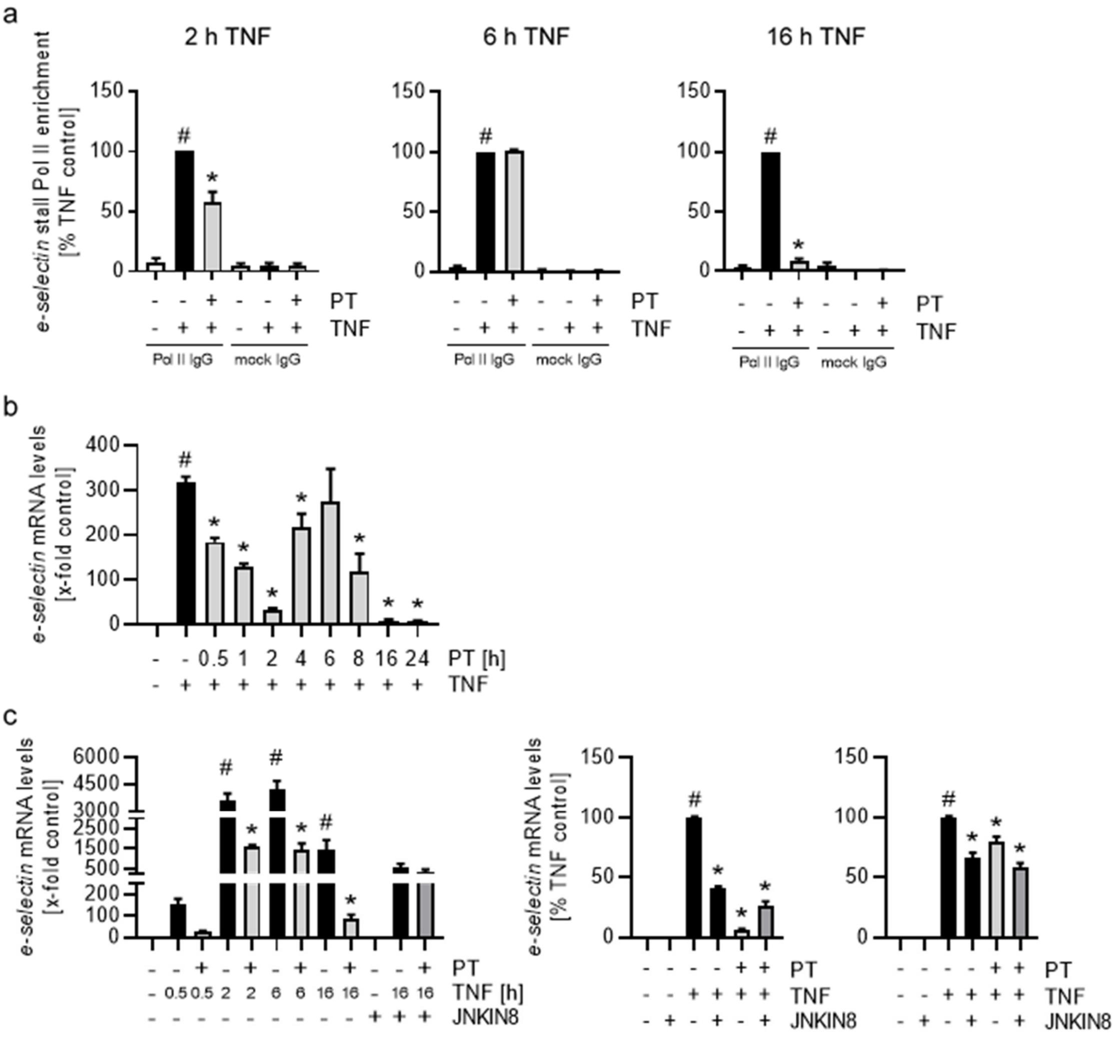

Supplemental Figure S17. Pretubulysin induces a time-dependent decrease of RNA polymerase II enrichment in the *e-selectin* stalling region and induces *e-selectin* mRNA levels at mean PT pre-treatment times, with the PT-induced down-regulation of *e-selectin* mRNA being sensitive to JNK inhibition. (a) Chromatin immunoprecipitation (ChIP) of the RNA polymerase II enrichment in the *e-selectin* stalling region. Confluent HUVECs were pre-treated with 300 nM of pretubulysin (PT) and activated with TNF (10 ng/mL) for different durations (2, 6, 16 h). ChIP was performed with an antibody against p65 or cJun and the respective mock antibody. Results were normalized on the background levels of a sequence of the *rpl13a* 3'-untranslated region. (b) qPCR analysis of the mRNA levels of E-selectin after different pretubulysin pre-treatment times. Confluent HUVECs were pre-treated with PT (300 nM) for different durations (0.5, 1, 2, 4, 6, 8, 16, 24 h) and activated with TNF (10 ng/mL) for 0.5 h. (c) qPCR analysis of the mRNA levels of E-selectin in dependency of different TNF induction and long-term JNKIN8 treatment time (left) and percentual representation of long-term TNF induction and JNK inhibition (middle) and short-term TNF induction and JNK inhibition (right). Confluent HUVECs were pre-treated with pretubulysin (300 nM) for 30 min and activated with TNF (10 ng/mL) for different durations (0.5, 2, 6, 16 h; c, left). For inhibition of JNK activity HUVECs were pre-treated with JNKIN8 (5 μM) for 30 min, treated with pretubulysin (300 nM) for further 30 min and activated with TNF (10 ng/mL) for 16 h (c, left/middle; long-term JNK inhibition) or 1 h (c, right; short-term JNK inhibition). Results were normalized on the respective *gapdh* levels. (n = 3) Data are expressed as mean ± SEM. # $p \leq 0.05$ versus control, * $p \leq 0.05$ versus TNF control.

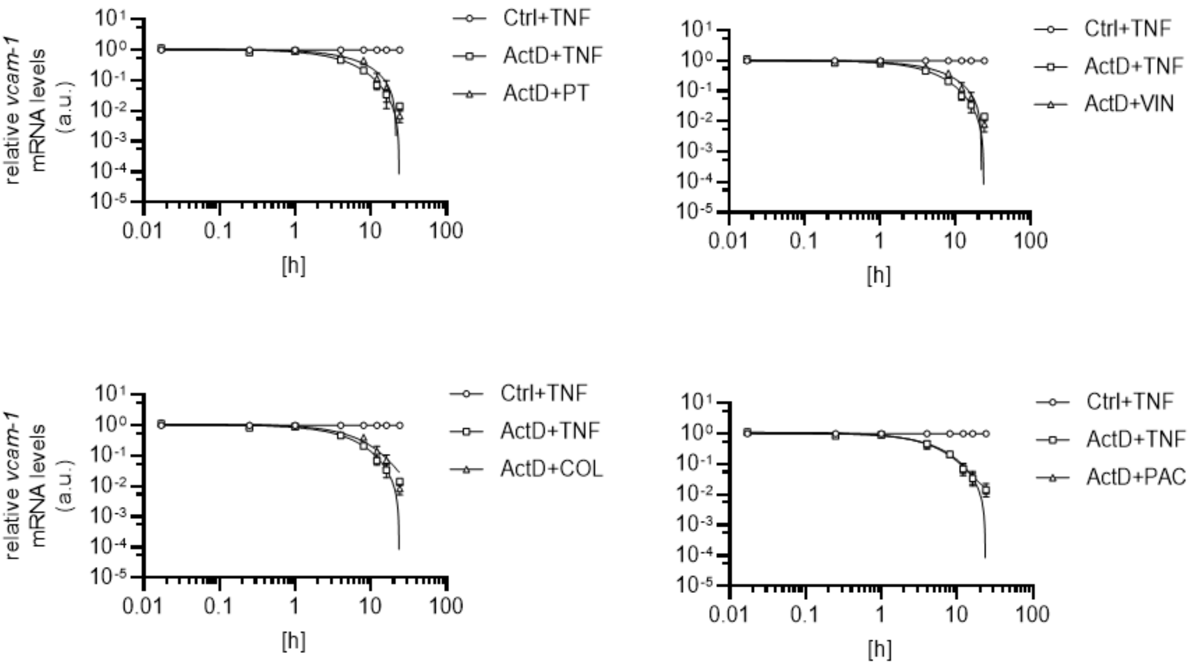

Half-life (h)	PT	VIN	COL	PAC
ActD+TNF	3.554 (0.9234)	3.554 (0.9234)	3.554 (0.9234)	3.554 (0.9234)
ActD+MTA	5.935 (0.8914)	5.715 (0.9596)	4.134 (0.9324)	3.044 (0.9642)

Supplemental Figure S18. Pretubulysin and the other destabilizing MTAs increase the half-live ($t_{1/2}$) of the *vcam-1* mRNA. qPCR analysis of the *vcam-1* mRNA levels after actinomycin D addition. Confluent HUVECs were treated with TNF (10 ng/mL) for 12 h. Actinomycin D (ActD; 2 µg/mL) was added to inhibit mRNA expression and 300 nM of pretubulysin (PT), vincristine (VIN), colchicine (COL) or paclitaxel (PAC) was added under continuous TNF treatment for different durations (5 min, 15 min, 1 h, 4 h, 8 h, 12 h, 16 h and 24 h). Results were normalized on the respective *gapdh* levels of the TNF control. The non-linear regression is based on the GraphPad Prism 'one-phase decay'. The corresponding R^2 values are shown in round brackets. (n = 3)

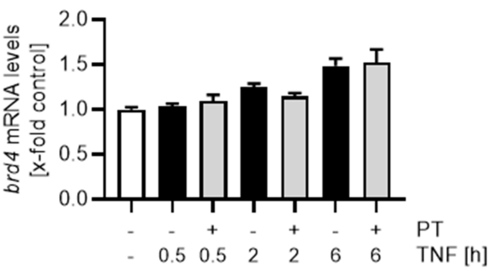

Supplemental Figure S19. Pretubulysin does not negatively impact the *brd4* mRNA levels. qPCR analysis of the *brd4* mRNA levels. Confluent HUVECs were pre-treated with pretubulysin (PT; 300 nM) for 30 min and activated with TNF (10 ng/mL) for different durations (0.5, 2, 6 h). Results were normalized on the respective *gapdh* levels. (n = 3)

Supplementary Table S1. Primers used for qPCR analysis of the relative mRNA levels (material and methods 2.10).

Gene	Forward (5′-3′)	Reverse (5′-3′)
icam-1	CTG CTC GGG GCT CTG TTC	AAC AAC TTG GGC TGG TCA CA
vcam-1	CCA CAG TAA GGC AGG CTG TAA	GCT GGA ACA GGT CAT GGT CA
e-selectin	AGA TGA GGA CTG CGT GGA GA	GTG GCC ACT GCA GGA TGT AT
NFκB-p65	GGG GAC TAC GAC CTG AAT GC	GAT CTT GAG CTC GGC AGT GT
NFκB-p105	CAT CCC ATG GTG GAC TAC CT	GGG CAT GCA GGT GGA TAT TT
AP-1-cjun	ATC AAG GCG GAG AGG AAG CG	TGA GCA TGT TGG CCG TGG AC
brd4	AAT AGC AGC AAC AGC AAT GTG AG	CTT CCT CCG ACT CAT ACG TGG
gapdh	CCA CAT CGC TCA GAC ACC AT	TGA AGG GGT CAT TGA TGG CAA

Supplemental Table S2. Primers used for ChiP-qPCR analysis (material and methods 2.13). For transcription factors, primer pairs were designed around the most promoter proximal transcription factor binding motifs. The primer pairs targeting the RNA polymerase II in the promoters of the CAMs (*Pol-prom.*) were designed around the position of the most promoter proximal TATA-box. For experiments regarding the stalling behavior of the RNA polymerase II and the enrichment of Brd4 (*Pol/Brd4-stall*), the same primers were used, which encompass the non-translated regions downstream of the respective CAM promoter.

Gene/target	Forward (5′-3′)	Reverse (5′-3′)	Amplicon size (bp)
icam-1-p65	GGA AAC GGG AGG CGT GG	GAG AGG GTC ATC CTC CCT CG	122
vcam-1-p65	GAA CTT GGC TGG GTG TCT GT	AAG GGT CTT GTT GCA GAG GC	94
e-selectin-p65	TAC GAT ATA AAG GCA TGG ACA AAG G	GGA GGG ATT GCT TCC TGT GAA	175
IκBa-p65	GAG TTT CTC CGA TGA ACC CCA	GGC GCC CTA TAA ACG CT	155
icam-1-cJun	ACA ATA ACA GTC TCC ACT CTC CG	AGG GCG ATG ACC CCG	150
vcam-1-cJun	GTT GAT GTT TGT TGC TAA AAG AAC T	ACG ACT ATG CCA TGT GAA TTG AT	196
e-selectin-cJun	TAC GAT ATA AAG GCA TGG ACA AAG G	GGA GGG ATT GCT TCC TGT GAA	175
icam-1-Pol/Brd4-stall	ATA AAG GAT CAC GCG CCC CA	CTT CGG AGA ACT GGC CCG AC	178
vcam-1-Pol/Brd4-stall	AAA GCA CAG ACT TTC TAT TTC ACT C	TTT GAG GCT CCA AGG ATC ACG	183
e-selectin-Pol-stall	TCC TAT AAA AG GGC CTC AGC CG	GCC CTT ATA AAG CGT TCT GCA CT	168
icam-1-Pol-prom.	GGG AAG GCG CGA GGT TT	CGG GGC TTC GGA GAA CTG	244
vcam-1-Pol-prom.	GAG GAG CTT CAG CAG TGA GAG	GAG GCC CGA TGC AGA TAC C	186
e-selectin-Pol-prom.	CCC GGG AAA GTT TTT GGA TGC	AGC GTT CTG CAC TTA CCG TT	244
rpl13a 3-UTR	CCA GGT CTC CAC GCT AAA CA	AAT CTT GGC ATC CAA CCG CA	99
